# Depression Symptoms in Patients with Diabetic Peripheral Neuropathy

**DOI:** 10.1900/RDS.2020.16.35

**Published:** 2021-05-01

**Authors:** Rahab Marhoon Alghafri, Alfred Gatt, Cynthia Formosa

**Affiliations:** Faculty of Health Sciences, University of Malta, Malta.

**Keywords:** diabetes, diabetic foot, peripheral neuropathy, depression, mood, screening

## Abstract

**AIM:**

The study aimed to investigate the possible relationship between diabetic peripheral neuropathy (DPN) and the development of depressive symptoms in patients with type 2 diabetes mellitus (T2D).

**METHODS:**

A comparative non-experimental study was conducted. Ninety-five T2D individuals aged 65 years and more were recruited. The sample was divided into two groups: 50 participants with T2D and without DPN and 45 participants with T2D and DPN. The Patient Health Questionnaire 9 (PHQ-9) was used to collect information about low mood and depression symptoms in the subjects recruited.

**RESULTS:**

Participants with DPN recorded higher scores on PHQ-9 than those with T2D only. The mean PHQ-9 score for the DPN group (6.09) was significantly higher than that for the T2D only group (2.24) (p < 0.001). Participants with DPN were more likely to have mild to moderate or moderately severe low mood and depression symptoms than T2D only participants who exhibited minimal to no low mood and depressive symptoms.

**CONCLUSIONS:**

The association between DPN and depression is confirmed by this study, with significant depressive symptoms found in patients with neuropathy when compared to diabetes patients with no neurological complications. It is therefore important that discomfort and emotional problems caused by DPN should be taken seriously and addressed closely in the management of DPN in order to prevent depression. Also, a change in screening practices to identify patients with diabetes and depressive symptoms is required.

## 1 Introduction

Depression and diabetes have surely ranked amongst the defining epidemics of the 21^st^ century, given the current rise in the prevalence rates of both these conditions in the world [1]. It can be observed that the prevalence rates of both conditions rise uniformly. Therefore, it is conceivable that there could be an interaction or dependency between these two conditions when one is present, leading to additional morbidity and a higher mortality in patients living with diabetes. However, the specific relationship between depression and diabetic peripheral neuropathy (DPN) remains unclear. Furthermore, neuropathic pain may cause the general health status of patients with diabetes to deteriorate [2].

The most common mental disorder is low mood leading to depression, which is one of the mental disorders that can be found in patients with diabetes [3]. Low mood and depression is defined as having signs of sadness, feeling of anxiety or panic, worry, low self-esteem, tiredness, anger, frustration, thoughts about death, losing or gaining weight, and alteration in activity and sleep pattern for more than two weeks. Depressed individuals show at least four of the aforementioned signs and symptoms [4]. Depression is classified as a very common and serious medical condition [5].

Diabetes can aggravate the symptoms of depression, and depression increases the risk of aggravating type 2 diabetes mellitus (T2D) [6]. This is due to the link between depression and poorly controlled health behavior, including low physical activities, smoking, and high food consumption which are directly linked to obesity and poor glucose control [7]. The association between depression and chronic conditions has been confirmed by several studies [8], and confirmed to lead to low quality of life [9]. Although many studies indicated the association between poor or low control of diabetes and depression, appropriate attention as given to other chronic health condition ist still lacking [10]. This study sought to investigate the association between DPN and symptoms of depression by measuring mood symptoms in participants with T2D and without DPN and comparing them with the mood symptoms in participants with T2D and DPN.

## 2 Methods

### 2.1 Study design

We performed a non-experimental, comparative, quantitative study in a diabetes primary care setting. The University of Malta Research Ethics Committee approved this study. After obtaining informed consent, patients with T2D were categorized into 2 groups using convenience sampling. Patients who satisfied the following inclusion criteria were recruited as follows:

1. Group 1: 50 participants with T2D and no DPN
2. Group 2: 45 participants with T2D and DPN

The following inclusion criteria were designed for the study: Male and female participants aged 60 years or more. The participants needed to give signed informed consent.

Participants were excluded from the study if they:

- Presented with type 1 diabetes mellitus
- Had other comorbidities that are chronic in nature such peripheral vascular disease (PVD), spinal canal stenosis, or arthritis
- Had a history of foot ulceration or antidepressant medication

Participants who agreed to participate were interviewed once during the study. Demographic data were recorded, including gender, age, level of education, medications, and their latest HbA1c level. The participants were asked to respond to the questions of the Patient Health Questionnaire 9 (PHQ-9) [11].

### 2.2 Assessment of diabetic peripheral neuropathy

Prior to the start of the interview, participants were assessed for peripheral neuropathy symptoms by a state registered podiatrist using the 10g monofilament and the 128 Hz tuning fork. Participants with a positive test for neuropathy were included in Group 2. Neurological assessment was performed in a quiet room by the same investigator to ensure repeatability.

The first tool used in this study was the 128 Hz tuning fork. This was applied first by striking the tuning fork so that it vibrated appropriately without creating audible humming. The tuning fork was then placed on the medial border of the hallux after the patient had been instructed to close the eyes. The participant was instructed to inform the practitioner of the type of sensation perceived and when exactly the vibrating sensation had ceased. The examiner certified the absence of vibration by placing the tuning fork on the dorsal aspect of the bony prominence of the patient’s own thumb. Normally, the practitioner is able to feel vibration persisting for at least 10 seconds after the participant’s perception has ceased, which was considered to be normal. The test was performed on both feet. For a diagnosis of neuropathy, the patient had to be unable to feel a buzzing sensation when the vibrating tuning fork was applied and at the same time pinpoint the moment that the tuning fork stopped vibrating. The patients who managed to satisfy these criteria were given the score ‘absent’. If the patient was able to feel the vibrating stimulus on the hallux then the patient was given the score of ‘present’.

The 10g Semmes-Weinstein monofilament was used to identify peripheral sensory neuropathy, as previously described by Boulton [12]. The 5-point test was used. The plantar region of the hallux and third digit together with the first, third, and fifth metatarsal heads were used for testing. With the eyes closed, the patient reported to the investigator when he or she could feel the monofilament. Inability to feel the 10 g of pressure was considered to be indicative of peripheral neuropathy.

### 2.3 Study tool for the measurement of low mood and depression

The early detection of low mood symptoms is important to prevent depression, since the literature suggests that low mood lasting for two weeks or more is a symptom of depression [13]. There are several scales used to test for depression, including the Hospital Anxiety and Depression Scales (HADS) [14], Patient Health Questionnaire 2 (PHQ-2) [11], and Patient Health Questionnaire 9 (PHQ-9) [15], amongst others.

For the purpose of this study, the Patient Health Questionnaire 9 (PHQ-9) was utilized to collect the information about low mood and depression symptoms in the subjects recruited. The PHQ-9 is an easy to use, self-rating tool that can be used alone or after using the PHQ-2 questionnaire to assess low mood and depression symptoms [11]. It has been validated to be used clinically [15], exhibits a sensitivity rate of 61% and specificity of 94% in adults, and requires no permission from the authors to be used in the clinical setting [11]. It can be used to make a tentative diagnosis of depression in at-risk populations, such as those patients with coronary heart disease or those following stroke. This tool has been validated for use in primary care settings [16]. Validity has been assessed against an independent structured mental health professional (MHP) interview. PHQ-9 score ≥10 had a sensitivity of 88% and a specificity of 88% for major depression [11]. It can even be used over the telephone [17]. Studies found that the PHQ-9 is also useful for screening for depression in psychiatric clinics [18].

The PHQ-9 questionnaire consists of 9 questions with four answer options:

1. “Not at all” scored as 0
2. “Several days” scored as 1
3. “More than half the days” scored as 2
4. “Nearly every day” scored as 3

The participants are requested to select one of the four response options for each of the 9 questions. The final PHQ-9 score indicates the severity of low mood or depression [11]. The interpretation of the total score of the PHQ-9 is classified into five subgroups. The minimum score that can be recorded is 0 and the maximum score is 27. The PHQ-9 classification groups are classified as follows:

- Score 1-4: Minimal depression
- Score 5-9: Mild depression
- Score 10-14: Moderate depression
- Score 15-19: Moderately severe depression
- Score 20-27: Severe depression

The results of the PHQ-9 interview may be used to make a depression diagnosis according to the criteria of the Diagnostic and Statistical Manual of Mental Disorders, 4^th^ edition (DSM-IV), which takes less than 3 minutes to complete. The total score from all 9 answers from the PHQ-9 aims to predict the presence and severity of depression. Primary care providers use the PHQ-9 to screen for depression in patients. A provisional diagnosis of a major depressive disorder can be made by using answers to PHQ-9 questions to fulfil the diagnostic criteria of DSM-5. According to DSM-5, a major depressive disorder is likely if 5 or more of the 9 symptoms are present for “most of the day, nearly every day” in the past 2 weeks and one of the symptoms is depressed mood or little interest or no pleasure in doing things (questions 1 and 2 on the PHQ-9). Any degree of suicidal thoughts counts toward these criteria. The symptoms must also cause significant distress and loss of function, and the symptoms must not be better explained by substance use or another medical or psychiatric condition.

“Other” depression is diagnosed if there is significant impairment and/or distress in major areas of functioning, but the full criteria for any specific depressive disorder are not met. Clinicians may also use the PHQ-9 to evaluate treatments given for depression. A change of PHQ-9 scores to less than 10 is considered a “partial response” to treatment and a change of PHQ-9 score to less than 5 is considered to be “remission.” [19].

### 2.4 Statistical analysis

All data were recorded on a spreadsheet designed in Microsoft Excel to assort the information required for interpretation of the results. The resulting data are expressed as mean ± standard deviation (SD). Descriptive statistics were used to characterize clinical variables of the patients studied. These data were analyzed by utilizing the IBM SPSS (Statistical Package for Social Sciences) program.

The overall scores of low mood and depression symptoms were obtained and descriptive analysis was performed based on the main study variables of both participant groups. Mean values for gender, age, level of education, treatment of diabetes, other medication intake, presence of neuropathy, HbA1c level, and duration of neuropathy were recorded. The Shapiro-Wilk test was used to test normality distribution of PHQ-9 scores for both groups. The non-parametric Mann-Whitney test was used to compare mean PHQ-9 scores between the two groups since the data were not distributed normally. The chi-square test was used to investigate the association between the PHQ-9 score classification (none or minimal, mild, moderate, moderately severe depression) and group (T2D, DPN).

## 3 Results

A total of 95 participants, 52 males and 43 females were recruited in this study. The number of participants with T2D only and participants with T2D and DPN were 50 and 45, respectively. The mean age of the study population was 77.55 years (SD 6.212). Most of the participants reported to have secondary level education (n = 50), while a few reported to have tertiary level of education (n = 4). Mean HbA1c for Group 1 was 5.9% (SD 0.85), while mean HbA1c for Group 2 was 8.2% (SD 1.74). The mean duration of neuropathy for Group 2 was 5.9 years (SD 4.04).

### 3.1 Individual answers to the PHQ-9 questionnaire

Tables 1 and 2 below include individual answers to the PHQ-9 questionnaire from both study groups. It is obvious from the percentages reported that participants with DPN reported more depressive symptoms than those with T2D only.

**Table 1.** Answers from participants with T2D only (Group 1, n = 50)

Questions from PHQ-9	Not at all	Several days	More than half the days	Nearly ever y day
1. Little interest or pleasure in doing things	82	14	2	2
2. Feeling down, depressed, or hopeless	84	12	4	0
3. Trouble falling or staying asleep, or sleeping too much	68	20	4	8
4. Feeling tired or having little energy	58	32	10	0
5. Poor appetite or over eating	80	12	8	0
6. Feeling bad about yourself or your family	92	6	2	0
7. Trouble concentrating on things, such as reading the newspaper or watching TV	96	2	2	0
8. Moving or speaking so slowly that other people could have noticed or the opposite	84	12	2	2
9. Thoughts that you would be better of f dead or hur	98	0	2	0

**Legend:** All numbers are percentages (%).

**Table 2.** Answers from participants with DPN (Group 2, n = 45)

Questions from PHQ-9	Not at all	Several days	More than half the days	Nearly every day
1. Little interest or pleasure in doing things	55.55	17.77	11.11	15.55
2. Feeling down, depressed, or hopeless	53.33	20	17.77	8.88
3. Trouble falling or staying asleep, or sleeping too much	46.66	26.66	15.55	11.11
4. Feeling tired or having little energy	40	24.44	13.33	22.22
5. Poor appetite or over eating	42.22	24.44	24.44	8.88
6. Feeling bad about yourself or your family	66.66	20	11.11	2.22
7. Trouble concentrating on things, such as reading the newspaper or watching TV	80	11.11	6.66	2.22
8. Moving or speaking so slowly that other people could have noticed or the opposite	77.77	11.11	8.88	2.22
9. Thoughts that you would be better	93.33	4.44	2.22	0

**Legend:** All numbers are percentages (%).

### 3.2 Difference in mean PHQ-9 scores between the study groups

The mean PHQ-9 score for the DPN group (6.09) was significantly higher than the mean PHQ-9 score for the T2D group (2.24) (p < 0.001). Table 3 below demonstrates the statistical difference in mean PHQ-9 scores between the study groups.

**Table 3.** Mann-Whitney test - difference in PHQ-9 scores between the study groups

Group	Sample size	Mean PHQ-9	SD	P-value
Group 1 (T2D only)	50	2.24	2.631	0.000
Group 2 (T2D and DPN)\	45	6.09	4.804	

### 3.3 Measuring the strength of the association

**Figure 1.**
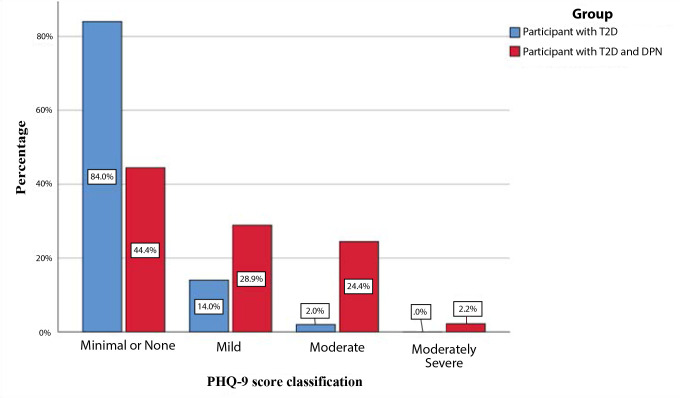
Percentages of PHQ-9 score classifications for each group.

The chi-square test was used to investigate the association between the PHQ-9 score classification (none or minimal, mild, moderate, moderately severe) and groups (T2D vs. DPN). Participants in Group 1 were more likely to have minimal to no low mood or depression symptoms than those in Group 2. On the other hand, participants in the DPN group were more likely to have mild to moderate or moderately severe depression symptoms than their counterparts in the T2D only group (X2(3) = 18.729, p < 0.001, see Table 4 and Figure 1).

**Table 4.** Percentages of PHQ-9 score classifications for each study group

PHQ 9 Score Classifications -		Group		Total
		Participants with T2DM	Participants with DPN	
Minimal or None	Count	42	20	62
	Percentage	84.0%	44.4%	65.3%
Mild	Count	7	13	20
	Percentage	14.0%	28.9%	21.1%
Moderate	Count	1	11	12
	Percentage	2.0%	24.4%	12.6%
Moderately Severe	Count	0	1	1
	Percentage	0.0%	2.2%	1.1%
Total	Count	50	45	95
	Percentage	100.0%	100.0%	100.0%

**Legend:** X2(3) = 18.729, p < 0.001

## 4 Discussion

Based on our data, this study has demonstrated that depression and anxiety disorders exist more frequently in patients with diabetes and DPN. Therefore, clinicians should pay attention to the fact that low mood and depression are common in patients with diabetes presenting with DPN as these conditions can complicate conditions and impair outcomes if overlooked and not addressed simultaneously in the clinical scenario. Patients should be asked about discomfort caused by DPN and emotional problems associated with DPN, and these problems should be addressed in a timely manner in the management of diabetes and DPN in order to prevent severe depressive symptoms in these patients. Depression is a severe state of discomfort and its prevalence rates are increasing in the 21^st^ century. According to the WHO, unipolar depressive disorders were ranked as the third leading cause of the global burden of disease in 2004 and will move into the first place by 2030 prospectively [20].

Depressive symptoms need to be paid attention, especially amongst high-risk populations with many comorbidities such as diabetes. Thus, routine screening for depression and anxiety in patients presenting with DPN is recommended. This is not standard practice amongst healthcare professionals at both primary and secondary care facilities to date [21], even though the American Diabetes Association has recommended that patients with diabetes, especially those with poorly controlled blood glucose, should be screened for depression [22]. Thus, a call for change in screening practices for patients with diabetes is necessary.

Healthcare professionals should be familiarized with the PHQ-9 questionnaire and other similar screening tools and use them routinely in their practice as an adjunct to normal consultation. These questionnaires are easy to use, not time-consuming, and are efficient and reliable in detecting patients with depressive disorders. If low mood or depression symptoms are detected, a prompt treatment plan should be implemented, including pharmacologic or nonpharmacologic options and prompt referral to appropriate healthcare professionals.

As a limitation of this study, the sample size was relatively small since it was difficult to find participants with DPN without a history of previous ulcer and without a current ulcer and no other diabetes-related complications such as PVD. A larger number of participants may provide more strength. It is therefore greatly recommended that further studies are performed with larger samples, given that mental health issues are currently reported to be one of the major global health issues.

Another limitation of this study may be that the modalities used in this study to diagnose DPN may not be sensitive enough to detect early neuropathy and small-fiber impairment. Thus, patients with early or advanced neuropathy could have been included in this study. However, the 10g monofilament and the 128 Hz tuning fork procedures are standard clinical tools used in primary care clinics to diagnose DPN, and they are very reliable

Patient compliance in answering the questions of the PHQ-9 questionnaire could also have imposed a limitation to the study findings. Since the questionnaire relies on patient self-reporting, all answers were verified by the clinician, and a definitive diagnosis was made based on clinical experience, taking into account how well the patient understood the questionnaire and other relevant information from the patient. For instance, participants found it difficult to answer question 9 which refers to “thoughts that you would be better off dead, or of hurting yourself” as some patients found it hard to express or estimate their feelings to give a correct answer. To overcome this difficulty, participants were given further explanation and examples for each question to understand better the question asked.

## 5 Conclusions

Depression seems to be a frequent comorbid condition in diabetes patients with DPN. Thus, patients with neurological symptoms caused by neuropathy are at high risk for depression. Therefore, discomfort caused by DPN and the emotional problems associated with DPN expressed by the patient should be addressed in the management of DPN in order to prevent depression in patients with diabetes.

Finally, we recommend a more widespread dissemination of this information through continuing medical education programs and other relevant means to improve outcomes in this population. More studies are needed to test the usefulness of the screening for depressive disorders amongst T2D patients with DPN.
